# Therapeutic potential of autophagy in immunity and inflammation: current and future perspectives

**DOI:** 10.1007/s43440-023-00486-0

**Published:** 2023-04-29

**Authors:** Hui Zuo, Cheng Chen, Yalian Sa

**Affiliations:** 1 Department of Pharmacology, The First People’s Hospital of Yunnan Province, Kunming, 650032 Yunnan Province China; 2 Department of Pharmaceutical Science, The Affiliated Hospital of Kunming University of Science and Technology, Kunming, 650032 Yunnan Province China; 3 Institute of Clinical and Basic Medical Sciences (Yunnan Provincial Key Laboratory of Clinical Virology), The First People’s Hospital of Yunnan Province, Kunming, 650032 Yunnan China

**Keywords:** Autophagy, Inflammation, Disease, Chemoprevention, Cancer

## Abstract

Autophagy is recognized as a lysosomal degradation pathway important for cellular and organismal homeostasis. Accumulating evidence has demonstrated that autophagy is a paradoxical mechanism that regulates homeostasis and prevents stress under physiological and pathological conditions. Nevertheless, how autophagy is implicated in immune responses remains unclear. It is well established that autophagy bridges innate and adaptive immunity, while autophagic dysfunction is closely related to infection, inflammation, neurodegeneration, and tumorigenesis. Therefore, autophagy has attracted great attention from fundamental and translational fields due to its crucial role in inflammation and immunity. Inflammation is involved in the development and progression of various human diseases, and as a result, autophagy might be a potential target to prevent and treat inflammatory diseases. Nevertheless, insufficient autophagy might cause cell death, perpetrate inflammation, and trigger hereditary unsteadiness. Hence, targeting autophagy is a promising disease prevention and treatment strategy. To accomplish this safely, we should thoroughly understand the basic aspects of how autophagy works. Herein, we systematically summarized the correlation between autophagy and inflammation and its implication for human diseases.

## Introduction

Autophagy is a basic protective mechanism for cell homeostasis by degrading redundant proteins, lipids, and organelles [1]. This mechanism is tightly regulated, and exemplified by the autophagy-related (ATG) proteins identified in the yeast *Saccharomyces cerevisiae* [2]. ATG proteins regulate the intracellular apparatus responsible for the autophagosome's development, freight assortment, and dealing with the lysosomal compartment. Numerous ATG genes have been identified, and several orthologs have been recognized as autophagy controllers in eukaryotic cells [3]. Based on the transportation pattern of contents, autophagy is mainly classified into macroautophagy, microautophagy, and chaperone-mediated autophagy [4]. Generally, intracellular incorporation bodies can be transferred to the autophagic pathway and fused with lysosomes. This catabolic interaction can yield two outcomes: forestall tissue harm and infection or support homeostasis in distressing conditions. The autophagosome, a bilayer membrane structure, is framed in the cytoplasm during autophagy. Then, the autophagosome is fused with lysosomes to convey the substance into the organelle lumen, where they are broken down, and macromolecules are reused. Under physiological circumstances, autophagy occurs at a baseline level and controls homeostasis. Nonetheless, it might be triggered by exposure to starvation to provide supplementary substrates, improving cell survival. Hence, autophagy significantly controls body homeostasis [5]. Also, numerous stimuli, such as endoplasmic reticulum (ER) stress, safe cell enactment, oxidative stress, and disease, can induce autophagy [6, 7]. In the context of cancer, growing evidence corroborates that autophagy might simultaneously exert pro-tumor and antitumor functions. On the one hand, autophagy suppresses tumor growth and invasion by degrading oncogenic products and damaged organelles. On the other hand, it might improve tumor growth by reusing intracellular substrates [8]. Herein, we provide a comprehensive review of the roles of autophagy in human diseases and its underlying mechanisms.

## Autophagy-related pathways and their roles in inflammation

Inflammation is a complex biological process/defense mechanism that is triggered by external or internal stimuli and plays a crucial role in maintaining homeostasis [9]. Various inflammatory cells, especially neutrophils and macrophages, are rapidly recruited into the infection focus, activate adaptive immune responses, and release inflammatory cytokines and chemokines. Physical and chemical non-microorganism-related factors, such as ischemia–reperfusion injury, usually cause sterile inflammation. The co-occurrence of infectious and aseptic inflammation leads to the release of cytokines and chemokines, including tumor necrosis factor (TNF) and interleukin-1 (IL-1). Caspase-1 can cleave pro-IL-1β and pro-IL-18 into their active forms and exacerbate proinflammatory reactions. This cycle happens in particular protein stages, called inflammasomes, collected after proinflammatory cell reactions [10].

**Fig. 1:**
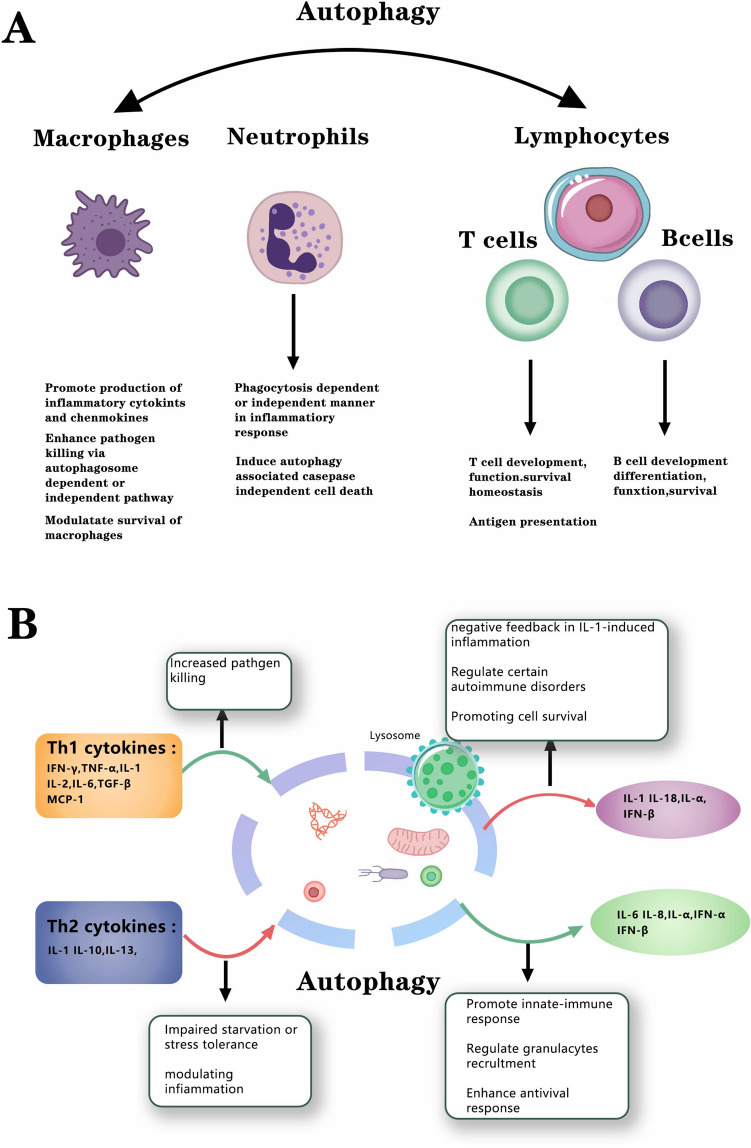
**A** Effects of autophagy in inflammatory cells. Autophagy influences inflammatory cells' development, homeostasis, and survival, including macrophages, neutrophils, T lymphocytes, and B lymphocytes. **B** Interactions of autophagy and inflammatory cytokines or chemokines. Autophagy can affect the secretion of cytokines such as Th1 cytokines I(FN-γ, TNF-α, IL-1, IL-2, IL-6, TGF-β, and MCP-1) and Th2 cytokines (IL-4, IL-10 and IL-13), as well as other cytokines (IL-1β, IL-18, IFN-a, IFN-β, and IL-8)

Canonical autophagy can catch various cargo to incorporate attacking organisms alongside their microorganism-related atomic examples (Pathogen-associated molecular patterns, PAMPs). This process can also remove dangerous cargo, including pathogens, unfolded proteins, and damaged organelles, and provide sources for biosynthesis. Autophagy can be classified as xenophagy (direct end of intracellular microorganisms), aggrephagy (expulsion of macromolecular totals or condensates), mitophagy (evacuation of mitochondria), endoplasmic reticulum-phagy (otherwise called reticulophagy), pexophagy (autophagy of peroxisomes), and lysophagy (expulsion of harmed lysosomes), for example. Mass autophagy comprises a discount reusing of supplements that can occur during a particular autophagy type via a bystander impact [11]. The evidence above indicates that autophagy is crucial in inflammatory reactions (Fig. 1A).

Considering that microorganisms, damaged proteins, and natural or inorganic germs can serve as inflammatory signals (PAMPs and damage-associated molecular patterns, DAMPs), the decomposable effect of autophagy is anti-inflammatory in cells capable of activating a cell-autonomous inflammatory reaction (Fig. 1B) that can affect the secretion of cytokines [12–14].

Additionally, autophagy shapes the inside of immune cells by regulating mitochondrial and ER homeostasis. Indeed, it regulates macrophage polarization (M1/M2), T-cell activation, and systemic inflammatory response [15–17]. Given its ability to regulate homeostasis, autophagy has been widely considered a significant controller of natural resistance to harmful substances [18]. Upon exposure to lipopolysaccharide (LPS), Beclin-1 is recognized by the Toll-like receptor 4-flagging complex and modified at K63 polyubiquitin chains, leading to the depolymerization of Beclin-1 and anti-apoptotic gene Bcl-2, resulting in the formation of Beclin-1-containing autophagy complexes.

This response is abrogated in the ultraviolet (UV) irradiation resistance-associated gene (UVRAG)-deficient mice that develop sepsis and oversecrete a particular arrangement of cytokines (e.g., IL-1β) cleaved by caspase-1. Autophagy concealment prompted by UVRAGFS articulation drives the NOD-like receptor thermal protein domain associated protein 3 (NLRP3) inflammasome because of its activity on mitochondria dynamics. Impressively, MitoQ treatment results in the over-activation of the NLRP3 inflammasome and mature IL-1β release, suggesting that mitochondria dysfunction causes autophagy-related activation of the inflammasome pathway. Thus, mitophagy represents a cytoprotective role in the convenient goal of inflammation. Autophagy deficiency in tissues can also prompt excessive reactions [19–21]. Inspired by these findings, we investigated other inflammation problems in UVRAGFS mice, such as dextran sulfate sodium (DSS)-induced colitis. UVRAGFS mice had aggravated colitis, which could be restored by NLRP3 hindrance. In summary, we can conclude that the bidirectional relationship between autophagy and the inflammasome might significantly explain inflammation-related pathologies.

### Secretory autophagy (SA) and inflammation

It has been well established that proteins can be secreted via conventional mechanisms through the recognition of N-terminal peptide. Alternatively, few cytosolic proteins lack N-terminal peptides and also could be secreted through distinct unconventional or “non-canonical” processes. Among these unconventional mechanisms, SA has been proven as an unconventional secretion pathway which was involved the autophagy pathway [22]. Recently, accumulating evidence demonstrated that SA possesses an important roles in the release of aggregation-prone proteins. Most importantly, SA was found to be implicated in the pathogenesis of neurodegenerative diseases, such as Parkinson's disease, and Alzheimer's disease, at least partially through releasing autophagy-based secretion of certain peptides [23]. For example, SA could regulate inflammation response through secreting mitochondria fragments, pathogen released from infected cells, or inflammatory cytokines [24, 25]. However, the large problem about the role of SA on inflammation still needs to be solved, such as, what aspects of the molecular machinery of secretory autophagy overlap with degradative autophagy? How does SA influence inflammation response, proinflammatory or anti-inflammatory?.

### Autophagy and cytokine

Cytokines are a kind of secreted proteins which mainly generated and released by macrophages and lymphocytes. Despite autophagy has been verified to participate in various inflammatory diseases, the functional roles of autophagy on the production and release of inflammatory cytokines is still fully elusive. Interestingly, autophagy might exhibit both pro-secretory and anti- secretory on cytokines. For example, Liang and colleagues [26] revealed that *Atg7* deletion could lead to increased generation of IL-1β and IL-18 without influence on the level of IL-6 and TNF-α. In contrast, blocking autophagy using autophagy inhibitor 3-methyladenine (3-MA) can effectively alleviate sepsis symptoms and reduce IL-6 and TNF-α production [27]. Furthermore, Atg5fl/fl lysM − Cre + mice demonstrated increased IL-1α, IL-12, and CXCL1 in lung tissue after M. tuberculosis infection, but the universal pro-inflammatory cytokines, such as IFN-γ, TNF-α, and IL-6 were not changed [28]. Collectively, this literature indicated that the role of autophagy on cytokine generation did not reach a consequence of general inflammatory stimulation, but it might implicate several specific mechanisms.

SA

## Autophagy influences various human diseases via inflammatory pathways

**Fig. 2:**
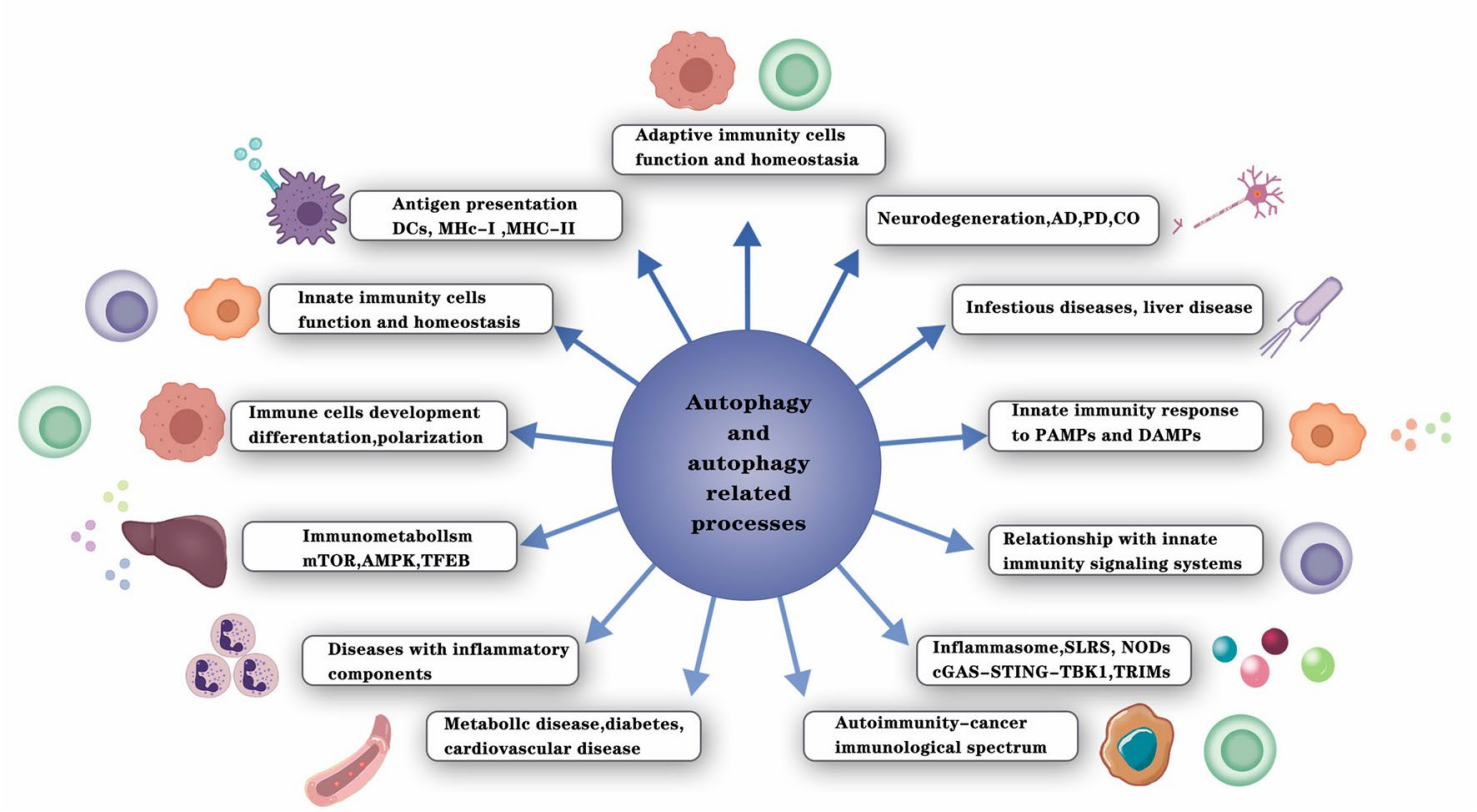
Summary of autophagy links with inflammation, innate immunity, adaptive immunity, and several diseases. Autophagy and autophagy-related processes affect immunometabolism, innate and adaptive immune cells, and immune responses (examples of diseases with inflammatory components affected by autophagy are presented)

Autophagy is related to various diseases, including neurodegeneration, infection, and cancer [29–31]. Here, we provide a comprehensive review of the regulatory role of autophagy in inflammation in different human diseases (Fig. 2).

### Cancer

Recently, autophagy has attracted increasing attention due to its critical role in tumorigenesis and tumor progression [32]. On a basic level, the cell-independent impact of autophagy exhibit tumor-promoting functions. FUN14 domain-containing 1 (FUNDC1) is a mitophagy receptor gene located in the external mitochondrial layer to eliminate damaged mitochondria via mitophagy under hypoxic conditions. Src can phosphorylate the LIR sequence of FUNDC1 under physiological circumstances; In contrast, hypoxia leads to the dephosphorylation, permitting it to bind with LC3 and promote mitophagy [33]. Many studies have shown that FUNDC1 is downregulated in hepatocellular carcinomas [34]. Hereditary FUNDC1 deletion can aggravate hepatocarcinogenesis and is associated with mitochondrial dysfunction, inflammasome activation, IL-1β release, and hepatocyte proliferation.

Furthermore, FUNDC1 supports hepatocarcinoma development by affecting mitochondrial function and metabolic reprogramming on malignant cells. Surprisingly, numerous significantly safe reconnaissances are similar to basic reprogramming cancer-related macrophages in hepatocellular carcinoma (HCC) and melanoma animal models, also initiated to growth stifling traditionally enacted aggregates through lysosomal capacity restraint and TFEB action. Besides, changing to glycolysis can diminish regulatory T cells and upgrade the anti-cancer action of infiltrating IFN-gamma (IFN-γ)-creating cytotoxic CD8^+^ T cells [35]. A metabolomic analysis demonstrated that cytotoxic CD8^+^ T cells undergo glycolysis in tamoxifen-induced Atg14l, Atg5, or Atg161 mice to inhibit the growth of mammary tumor [36]. Autophagy is involved in T-cell exhaustion in the recurrently infected liver [37], increasing immune tolerance due to the activation of administrative T cells [38].

Previous research has demonstrated that major histocompatibility class I (MHC-I) glycoproteins are dramatically reduced in pancreatic ductal adenocarcinoma cells (PDACs) and are important for immunotherapy response [39]. This phenomenon happens via the lysosomal degradation of MHC-I, conceivably through a standard autophagic system, and is not connected to a non-accepted LC3-associated phagocytosis (LAP) or LC3-associated endocytosis (LANDO) course precluded in Run domain Beclin-1 interacting and cysteine-rich containing protein (RUBICON) decrease tests. However, LAP in tumor-associated macrophages can exert an immunosuppressive function [40]. In B16F10 xenograft melanoma, deleting Rbcn (a distinctive LAP component) in myeloid cells effectively alleviates tumor growth and metastasis. Becn1 and Atg 5 have a similar trend, which was not observed for Fip200 or Atg14l.

Moreover, the stimulator of interferon genes (STING)–IFN signaling pathway becomes activated and promotes the activation and infiltration of cytotoxic T cells into the tumor core. A recent study evidenced that radiotherapy could induce grounded “abscopal” (distant tumor) benefits in mammary carcinoma TS/A cells when Atg5/ or Atg7/TS/A clones were used or upon chloroquine treatment. Besides, chloroquine effectively suppresses mitophagy and enhances radiotherapy-related immune responses via the mitoDNA-STING-cGAS-type I IFN pathway. Considering that type I IFN plays a significant role in malignant growth [41], and that cGAS potentiates insusceptible designated spot barricade in programmed cell death ligand-1 (PDL-1) treatment [42], autophagy (or LAP) with type I IFN reactions associated with STING-TBK1 represent a potential target for cancer treatment.

New evidence has demonstrated that autophagy can repress malignant growth by coordinating its aggravation and vulnerability, notwithstanding its cell-independent roles of tumorigenic capacities. While cancer advances inflammation, autophagy induces antigen exposure and increases tumor vulnerability. Although disease cells can escape immunosurveillance by tuning down autophagy, chemotherapeutic drugs with immunogenic properties might increase immune responses against tumor cells by activating autophagic cell demise. Understanding the multifaceted and complex connections inside this carriage and how they are impacted via autophagy-inducing medications should elucidate ways to treat malignant tumor growth adequately. The autophagic apparatus is associated with some types of tumors. There are two main connections, one during malignant growth and the other during disease treatment. Some studies have shown that allelic Beclin 1^+/−^ was associated with increased weakness in various malignancies, including ovarian, prostate, and thoracic tumor [43]. Meanwhile, heterozygous Beclin 1^+/−^ mice experience unconstrained tumorigenesis. Likewise, unconstrained tumorigenesis is associated with the supply of other ATGs, such as UVRAG and ATG4 [44]. Finally, autophagy inhibition can be perceived as a pathway to inhibit malignant tumor growth, and its diminished action might be linked with the progression of human diseases.

### Cardiovascular diseases

Autophagy participates in several cardiovascular illnesses, such as atherosclerosis and cardiovascular breakdown, by regulating immune responses [45–47]. Cholesterol accumulation in lysosomes can induce lysosomal dysfunction and activate downstream NLRP3 inflammasome [48]. Interestingly, a high-fat diet is usually connected with mTOR activation and subsequent suppression of mitophagy and autophagy in macrophages, besides plaque instability.

### Infectious diseases

Xenophagy is joined by an equivalent or more significant autophagy activity in regulating inflammation and tissue damage. Consistent with the Mtb disease, xenophagy is a strategy synergic to anti-microbials [49, 50], in which the non-attendance of autophagy-related genes is overwhelmed by aggravation. Increased lung damage is observed in Mtb-tainted mice with abnormal Atg5 in myeloid cells and whole-body Smurf1 mice [51]. Gal3 or Gal8 deletion increases the sensitivity to Mtb diseases, partly ascribed to MERi [52]. Excessive inflammatory reactions seen in the endless supply of specific ATGs can be protective in a few circumstances. For example, myeloid-specific Fip200, Atg5, and Atg7 upregulation raises basal respiratory inflammation and protects against influenzas or reactivation herpesvirus [53]. Recently, some studies demonstrated that autophagy is associated with SARS-CoV-2 infections since the coronavirus and autophagosome are double membrane structures with slight differences [54]. Additionally, CRISPR genomic screens in cells deficient in type I IFN-inducing frameworks have demonstrated that critical autophagy genes (TMEM41B and VMP1) participate in the cytopathic effect (CPE) of SARS-CoV-2, while several autophagy-related genes (ATG3, ATG5, ATG7, and ATG12) might suppress CPE [55, 56].

However, the reason for the high morbidity and mortality of coronavirus disease 2019 (COVID-19) is still elusive. Changes from asymptomatic infections to respiratory failure involve various organs and tissues [57]. Interestingly, the spike protein of severe acute respiratory syndrome coronavirus 2 (SARS-CoV-2) crosslinks with T cells or three distinct immunotypes [58]. Studies have also revealed contradictory findings during different infections: hereditary deficiency in Toll-like receptor 3 (TLR3)—and TANK-binding kinase 1 (TBK1)-mediated type I IFN signaling [161] are associated with resistant type I IFN signaling in severe COVID-19 infection patients [59]. Notably, TLR3 and double-stranded RNA (dsRNA) ligands do not induce autophagy, but TBK1 is relevant to autophagy [60, 61]. Hence, autophagy and type I IFN frameworks are connected. Interchanges among autophagy and type I IFN and how autophagy influences different signaling can promote or alleviate inflammation reactions when exposed to SARS-CoV-2. Therefore, the internal connections between autophagy and SARS-CoV-2 should be explored in greater depth.

### Crohn’s disease

Crohn’s disease (CD) is characterized by typical inflammatory bowel disorder from the dysfunction in self-acknowledgment of commensal stomach greenery with abandons in the intestinal mucosa [62]. The pathological mechanism for CD remains unclear, but some studies have uncovered three main pathways involved in CD: ATG16L1, insusceptibility-related GTPase family M (IRGM), and nucleotide oligomerization domain contain protein 2 (NOD2) [63]. We previously mentioned that ATG16L1 is fundamental for the legitimate extension of the separation layer. IRGM induces autophagy through IFN-γ, which prompted by bacterial infections [64]. In parallel, NOD2, an intracellular PRR of the NLR family, is responsible for communication in a predetermined number of tissues and cells that incorporates Paneth and monocyte-inferred cells. NOD2 initiates ATG16L1 to bacterial passage destinations, focusing on microscopic organisms for autophagic corruption [65]. Surprisingly, recent studies have demonstrated that NOD2 acts by recognizing bacterial ligand muramyl dipeptide (MDP), inducing autophagy in essential antigen-presenting cells and monocyte-derived dendritic cells (DCs). This peculiarity demands NOD2 and the NOD2 flagging arbiter RIPK-2, but not NALP3, a PRR that also perceives MDP. Different groups have found that NOD2-instigated autophagy requires autophagy proteins, including PI3K, ATG5, ATG7, and ATG16L. Moreover, NOD2-interceded antigen presentation demands autophagy and DCs in CD, while variations in NOD2 and ATG16L1 T300A are associated with diminished surface MHC II and are insufficient for inducing antigen CD4 + T cell reactions [66].

Some studies have proposed that ATG16L1 T300A variations decrease the autophagic freedom of intestinal microorganisms, such as disciple intrusive *Escherichia coli* or *Salmonella typhimurium*. A previous review also depicted that ATG16L1 changes in mice lead to irregularities pertinent to CD development and progression [67]. The authors found a strong hereditary association between the ATG16L1 mutation and a particular strain of intestinal norovirus infection. Besides vulnerability quality cooperation, this infection modifies the transcriptional mark of Paneth cells and exacerbates immune reactions in mice treated with the harmful substance dextran sodium sulfate by increasing TNFα and IFNγ production and commensal microbes.

Furthermore, macrophages from mice lacking ATG16L1 in hematopoietic cells produce more IL-1β after LPS injection or infection with painless intestinal microorganisms. These mice are profoundly delicate to sodium sulfate-instigated colitis, suggesting that enhanced production of pro-inflammatory cytokines by macrophages might promote gastrointestinal harm in ATG16L1-subordinate CD. Generally, decreased autophagy might change xenophagic bacterial flexibility, promoting cytokine production and extracellular discharge pathways and advancing CD pathogenesis [68].

### Parkinson’s disease

Parkinson’s disease (PD), one of the most common progressive neurodegenerative diseases, is characterized by the degeneration of dopaminergic neurons. Its etiology is portrayed by the occurrence of intracellular incorporations (namely Lewy bodies), which contains α-synuclein and ubiquitin proteins, autophagosomes, and harmed mitochondria. The most common PD type is irregular PD, albeit family heredity is relevant [69]. Moreover, many studies have demonstrated that various pro-inflammatory cytokines participate in PD pathogenesis, such as TNF-α, IL-4, IL-6, IL-10, and IL-1β [70]. Several factors, such as a-synuclein, can serve as DAMPs to recognize PRRs, and leucine-rich repeat kinase 2 (LRRK2) (from the RIPK family) and are involved in autophagy induction. Exogenous overexpression of a-synuclein leads to lysosomal damage and autophagy, which can be stimulated by tau protein related to Alzheimer's disease [71]. Exceptionally compelling is the framework comprising E3 ubiquitin ligase PRKN and PINK1 fit for driving mitophagy. However, in animal models, PINK1 and PRKN are not associated with PD. When Prkn^−/−^ or Pink1^−/−^ mice are exposed to several mitochondrial stresses (changes in mitochondrial DNA, hyperactivity), they develop increased inflammatory signaling and PD [72]. Blocking STING signaling can effectively alleviate these phenotypes in Prkn^−/−^ or Pink1^−/−^ mice, leading to mitophagy failure, mitochondrial DAMP delivery, and the cytosolic DNA detecting framework working together with STING to aggravate PD. Additionally, transformations might enhance α-synuclein levels and induce familial PD. Although autophagy is reversed by A53T overexpression, the overabundance of intracellular α-synuclein disables autophagy by restraining the small GTPase Rab-1A. Finally, increased α-synuclein expression can induce protein accumulation and diminish autophagy, decreasing mitophagy and increasing neuronal apoptosis.

### Alzheimer’s disease

Alzheimer’s disease (AD) is the most common neurodegenerative disorder and comprises a decline in thinking and independence in daily activities. Two classical AD hallmarks are the accumulation of p-tau protein and the deposition of Aβ plaques [73]. Previous studies in biopsied AD neocortexes indicated thick lysosomes and different autophagic vacuoles, including autophagosomes, amphisomes, multilamellar bodies, and autolysosomes, addressing “transitional” stages in autophagy movement [74]. These perceptions proposed that imperfections in autophagic development might be an overall element of AD pathology. For example, in AD, autophagy might be weakened by autophagosome corruption and autophagosome formation, albeit those effects might fluctuate according to genotype and diseased phase. Hereditary examinations have also demonstrated a few transformations that induce intriguing familial AD types, such as the mutation of amyloid antecedent protein (APP), presenilin-1, and presenilin-1–1 (PS) 1 and 2 [75, 76]. Also, autophagy might be downregulated during autophagosome development in AD patients. Compared to healthy individuals, AD patients show diminished Beclin-1 articulation, which might prompt disability in autophagic movement. Beclin-1 heterozygous knockout in mice that express the AD-related human APP leads to APP and Aβ accumulation and displays more extreme neurodegeneration contrasted with Beclin-1 WT mice. Additionally, pathogenic APP and tau are corrupted via autophagy. Consistently, 3-MA, a specific autophagy inhibitor, increments tau harmfulness, while rapamycin (autophagy inducer) diminishes tau effects in the cell [77]. Nonetheless, a previous review has shown that 3-MA or Beclin-1 knockdown could reduce Aβ disposition in neuroblastoma and glioma cells [78]. The discussion on the cytoprotective versus cytotoxic roles of autophagy in AD models might be clarified by assessing the autophagic motion and the level of lysosomal imperfection for each situation. Further examinations are expected to expand the role of autophagy in AD.

## Discussion

Autophagy was first identified as a key mechanism in cancer development. A cancer-suppressive role for autophagy corroborates this. At the same time, its hereditary inactivation, either by constitutive activation of the PI3K (Phosphatidylinositol 3-kinase)/AKT (AKT serine/threonine kinase) pathway through PI3K changes, AKT enhancements, or Phosphatase and Tensin Homolog (PTEN) misfortune, or by allelic loss of Beclin-1 or lack in Atg5, can also increase carcinogenesis. Interestingly, this idea has been challenged by some that propose that autophagy can support oncogenesis because it can assist growth cell endurance [79]. This might be related to autophagy’s critical role in tumorigenesis because they share similar molecular mechanisms. A growth silencer engaged with the upstream restraint of mammalian target of rapamycin (mTOR) flagging (PTEN, TSC1, and TSC2) turns autophagy on while mTOR activates oncogenes. For example, class I PI3K and Akt switch it off [80]. Besides, p53 and Death-associated protein kinase (DAPK) are associated with human malignant growth and control autophagy [81]. The cell oncogenes Bcl-2 and Bcl-XL are frequently upregulated in human diseases and repress autophagy by inhibiting Beclin-1. Besides, p62 overexpression (via autophagy-lysosome) promotes carcinogenesis via NF-κB flagging liberation, activating nuclear factor E2-related factor (Nrf-2), inducing ROS production, and leading to DNA damage. The contrast between these paradoxical features of autophagy makes the association with disease treatment more complex. Notwithstanding, it has been recommended that in the initial phases of malignant growth, quality control via autophagy, especially over genome upkeep, suppresses carcinogenesis. Autophagy might organize the support or passage of cells into the G0 stage and subsequently forestall the unconstrained proliferation of tumor cells. Conversely, autophagy might provide nutrition for cancer cells and assist their growth when suffering from metabolic pressure and oppose passing set off by chemotherapeutics [82].

Furthermore, autophagy promotes growth cell endurance in normal and cancer cells. Although autophagy can postpone apoptosis, cell passing ultimately restricts autophagy. Apoptosis ordinarily escapes growing cells, granting supported endurance, movement, and protection from treatment. The delayed pressure endurance managed by imperfect apoptosis occurs in cancer cells by either increased anti-apoptotic genes Bcl-2 and Bcl-xL or deficiency in pro-apoptotic genes Bax and Bak [83]. The shortfall of cell passing is insufficient to support the pressure endurance of growing cells. Thus, the pressure from glucose oxygen deprivation strongly enacts autophagy, upholding apoptotic cells' long-term endurance. Cancer cells evading apoptosis can also obtain nutrition via autophagy when they endure pressure for a long time and enter a torpid condition. They can leave torpidity to continue cell multiplication when the pressure is released and typical development conditions are reestablished [84]. Hereditary or pharmacologic concealment of autophagy advances cell demise by putrefaction in vitro and in vivo, which suggests that growing and quiescent cells use autophagy to keep up with endurance in distressing conditions [85]. Autophagy limits these hypoxic districts, where it upholds growing cell endurance. Oxygen-detecting hypoxia-inducible factors activate autophagy alongside other metabolic factors and favor angiogenesis pathways unaffected by cell variation to metabolic pressure. Autophagy induction in hypoxic areas might also hamper treatment due to proliferative cells that are resistant to treatment in these hypoxic areas. Hence, determining the cancer cell torpidity and recovery component and how to target this pathway to build novel anti-cancer strategies is essential. Currently, lysosomotropism specialists (e.g., chloroquine) that hinder motion via autophagy and forestall autophagic freight debasement can be used as an autophagy inhibitors against malignant growing cells [86].

On the other hand, autophagy can also effectively exhibit antitumor activity in some contexts, especially in focused growth cells or when blended with restorative mTOR hindrance. In this case, autophagy might improve endurance, conceivably subverting treatment. Besides, various strategies using 3-MA, chloroquine, or hereditary manipulation of autophagy-related genes have shown that autophagy hindrance might sharpen growing cells to death, acting on assorted cytotoxic specialists [87]. Also, a few Phase I/II clinical trials involving chloroquine or hydroxychloroquine with chemotherapy for treating hematological malignancies are available [88]. Moreover, proteasome inhibitors can effectively trigger autophagy. Mechanistically, proteins can be degraded via two classical pathways: autophagy–lysosomal and ubiquitin–proteasome pathways. Inhibiting the ubiquitin–proteasome pathway activates the autophagy–lysosomal pathway. For example, Bortezomib (an FDA-approved proteasome inhibitor) effectively enhances autophagy in colorectal cancer and myeloma cells [89, 90]. Consistently, proteasome hindrance in prostate malignant growing cells by NPI-0052 can act through autophagy by an eIF2α-subordinate component that controls ATG function [91]. The concurrent inhibition of the two systems can result in a more effective strategy against cancer cells than the restraint of either pathway alone, which should be tested in the future.

## Conclusion

In summary, this review provided a profound understanding of the relationship between inflammation and autophagy in various human disorders. Autophagy can assume fundamental roles in inflammatory diseases, infections, and carcinogenesis. A better comprehension of autophagy in different diseases has promising effects on developing improved treatments. Meanwhile, autophagy studies are still being conducted, although their relevance to digestion, stress reaction, and cell demise pathways is recognized. Consequently, this cycle and their related reactions might provide data on how the host reacts to exogenous microorganisms and endogenous particles created under pressure conditions, yet these occasions can be re-molded by different stimuli and cell types. Altogether, understanding how autophagy is regulated and directed, and the particularity related to cell utilization, requires further examination. It will be essential to characterize and portray sub-atomic and biochemical features associated with the intricate exchange among autophagy and different pathologies to advance novel approaches for patients with neurodegenerative diseases and infections. The field of autophagy in immunity and inflammation-related diseases continues to evolve in both fundamental and translational fields. In general, almost all human diseases possess an inflammatory component, which in turn provides a window of opportunity and a challenge to develop autophagy-based therapeutic strategies. Taken together, these data suggest autophagy as a primordial determinant of human disease, thus delineating autophagy‐modulating interventions as potential strategies to treat or alleviate phenotypic anomalies of the most common human illnesses, including cancer, infectious, neurodegenerative, and cardiovascular, et al. Considering the irreplaceable role of autophagy in the removal of the primary toxic entity causing disease and subsequently reducing the susceptibility to pro-death insults, which implying autophagy is a promising target mechanism from a therapeutic perspective. However, to achieve this goal, a better understanding of autophagy’s fundamental biology is urgently needed. Finally, various pre-clinical and clinical studies are needed to investigate the function of autophagy in several diseases.

## Data Availability

This article has no additional data.

## Abbreviations

ATG: Autophagy related
ER: Endoplasmic reticulum
TNF: Tumor necrosis factor
IL-1: Interleukin-1
PAMPs: Pathogen-associated molecular patterns
DAMPs: Damage-associated molecular patterns
LPS: Lipopolysaccharide
UVRAG: Ultraviolet irradiation resistance-associated gene
NLRP3: NOD-like receptor thermal protein domain associated protein 3
DSS: Dextran sulfate sodium
FUNDC1: FUN14 domain-containing 1
HCC: Hepatocellular carcinoma
IFN-γ: IFN-gamma
MHC-I: Major histocompatibility class I
PDACs: Pancreatic ductal adenocarcinoma cells
LANDO: LC3-associated endocytosis
RUBICON: Run domain Beclin-1 interacting and cysteine-rich containing protein
LAP: LC3-associated phagocytosis
PD-L1: Programmed cell death ligand-1
STING: Stimulator of interferon genes
CPE: Cytopathic effect
COVID-19: Coronavirus disease 2019
SARS-CoV-2: Severe acute respiratory syndrome coronavirus 2
TLR3: Toll-like receptor 3
TBK1: TANK-binding kinase 1
ds RNA: Double-stranded RNA
CD: Crohn’s disease
IRGM: Insusceptibility-related GTPase family M
NOD2: Nucleotide oligomerization domain contain protein 2 (NOD2)
MDP: Muramyldipeptide
DCs: Dendritic cells
PD: Parkinson’s disease
LRRK2: Leucine-rich repeat kinase 2
AD: Alzheimer’s disease
APP: Amyloid antecedent protein
PI3K: Phosphoinositide 3-kinase
AKT: AKT serine/threonine kinase
PTEN: Phosphatase and Tensin Homolog
mTOR: Mammalian target of rapamycin
DAPK: Death-associated protein kinase
Nrf-2: Nuclear factor E2-related factor
SA: Secretory autophagy
